# Genetic Inhibition of Mitochondrial Permeability Transition Pore Exacerbates Ryanodine Receptor 2 Dysfunction in Arrhythmic Disease

**DOI:** 10.3390/cells12020204

**Published:** 2023-01-04

**Authors:** Arpita Deb, Brian D. Tow, You Qing, Madelyn Walker, Emmanuel R. Hodges, James A. Stewart, Björn C. Knollmann, Yi Zheng, Ying Wang, Bin Liu

**Affiliations:** 1 Department of Biological Sciences, Mississippi State University, Starkville, MS 39762, USA; 2 Beijing Key Laboratory for Agricultural Application and New Technique, College of Plant Science and Technology, Bioinformatics Center, Beijing University of Agriculture, Beijing 102206, China; 3 School of Pharmacy, Division of BioMolecular Sciences, University of Mississippi, Oxford, MS 38677, USA; 4 Department of Medicine, Vanderbilt University School of Medicine, Nashville, TN 37232, USA

**Keywords:** CPVT, calcium signaling, EC-coupling, mitochondria

## Abstract

The brief opening mode of the mitochondrial permeability transition pore (mPTP) serves as a calcium (Ca^2+^) release valve to prevent mitochondrial Ca^2+^ (mCa^2+^) overload. Catecholaminergic polymorphic ventricular tachycardia (CPVT) is a stress-induced arrhythmic syndrome due to mutations in the Ca^2+^ release channel complex of ryanodine receptor 2 (RyR2). We hypothesize that inhibiting the mPTP opening in CPVT exacerbates the disease phenotype. By crossbreeding a CPVT model of CASQ2 knockout (KO) with a mouse missing CypD, an activator of mPTP, a double KO model (DKO) was generated. Echocardiography, cardiac histology, and live-cell imaging were employed to assess the severity of cardiac pathology. Western blot and RNAseq were performed to evaluate the contribution of various signaling pathways. Although exacerbated arrhythmias were reported, the DKO model did not exhibit pathological remodeling. Myocyte Ca^2+^ handling was similar to that of the CASQ2 KO mouse at a low pacing frequency. However, increased ROS production, activation of the CaMKII pathway, and hyperphosphorylation of RyR2 were detected in DKO. Transcriptome analysis identified altered gene expression profiles associated with electrical instability in DKO. Our study provides evidence that genetic inhibition of mPTP exacerbates RyR2 dysfunction in CPVT by increasing activation of the CaMKII pathway and subsequent hyperphosphorylation of RyR2.

## 1. Introduction

The release of calcium (Ca^2+^) from the intracellular Ca^2+^ store sarcoplasmic reticulum (SR) via the channel complex ryanodine receptor (RyR2) regulates the heartbeat [1]. Ca^2+^ is sensed by the myofilament contractile apparatus, translating the Ca^2+^ signal into the mechanical contraction and relaxation of the heart. The Ca^2+^ release process is tightly controlled in healthy hearts, but goes awry in diseased hearts due to dysfunctions of the Ca^2+^ handling proteins. For instance, genetic or acquired defects of the RyR2 channel complex have been shown to contribute to a range of cardiac disorders [1,2,3]. These defects typically make the channel hyperactive or leaky, thus, giving rise to aberrant Ca^2+^ release (ACR) [1], which is implicated in life threatening cardiac arrhythmias. Genetic mutations have been identified in RyR2 and its multiple accessory proteins that cause a genetic arrhythmia syndrome called catecholaminergic polymorphic ventricular tachycardia (CPVT) [2,4]. CPVT is a stress-induced arrhythmia disease that is triggered by elevated levels of catecholamines during exercise or emotional stress. However, patients do not exhibit the macroscopic structural remodeling that is often observed in structural heart disease, thus, making diagnosis particularly challenging [2,4].

Mitochondria, commonly known as the powerhouse of the cell, are also critically involved in Ca^2+^ signaling [5]. They sense the intracellular Ca^2+^ signal to regulate energy production and cell death. Ca^2+^ enters the mitochondria primarily through the mitochondrial Ca^2+^ uniporter (MCU), and leaves mainly via the mitochondrial Na^+^/Ca^2+^ exchanger (mNCX) [6,7,8]. Mitochondrial Ca^2+^ (mCa^2+^) influx and efflux needs to be finely balanced to maintain mCa^2+^ homeostasis and mitochondrial function. When overloaded with Ca^2+^, the permeability of the inner mitochondria membrane increases due to the opening of the mitochondrial permeability transition pore (mPTP), a meta-channel with an unknown molecular identity [9,10]. The sustained opening of mPTP leads to membrane potential collapse, metabolite loss, cessation in ATP synthesis, mitochondrial swelling, and eventually cell death [10] under pathological conditions. Indeed, it has been shown that the inhibition of mPTP opening using genetic or pharmacological approaches protects against mCa^2+^ overload-induced cell death in the setting of ischemia-reperfusion injury [11,12,13,14,15,16]. However, recent studies have proposed that the opening of the mPTP also plays a physiological role in mediating mCa^2+^ efflux, thus preventing mCa^2+^ overload [17,18]. While preventing the prolonged opening of mPTP and subsequent cell death may be beneficial, blocking the mPTP-mediated mCa^2+^ efflux could potentially lead to harmful effects.

When challenged with sustained ACR due to RyR2 dysfunction, mitochondria may rely more heavily on mPTP-mediated Ca^2+^ extrusion to avoid mCa^2+^ overload [19]. Indeed, in our previous report [19], we observed that the frequency of MitoWinks (an indicator of mPTP-mediated Ca^2+^ efflux) increased significantly in CPVT and another disease model also marked by RyR2 dysfunction. Thus, we generated a genetic model to inhibit mPTP opening in CPVT to test the hypothesis that the arrhythmia syndrome will be exacerbated. In this study, we thoroughly examined the consequence of genetic inhibition of mPTP in the CPVT model and also explored the mechanism underlying the exacerbated cardiac arrhythmia syndrome.

## 2. Materials and Methods

### 2.1. Generation of Mouse Models

We employed a classic CPVT mouse model of CASQ2 KO [20], which phenocopies CPVT caused by mutations in CASQ2, a protein that stabilizes RyR2 from the SR luminal side [21]. To examine the consequence of genetic inhibition of mPTP in the setting of CPVT, we generated a double knockout (DKO) model by crossbreeding the CASQ2^−/−^ mouse with a KO model missing CypD, an activator of mPTP [9]. Wild type (WT), CASQ2^−/−^ [20], CypD^−/−^, and DKO mice in the C57BL/6 genetic background were used in this study. The genotypes of the crossbred mice were confirmed with polymerase chain reactions (PCR; for CASQ2^−/−^ and CypD^−/−^) using tail DNA. Age- and litter-controlled mice, both male and female (aged 2–8 months), were utilized for experiments.

All animal procedures were approved by the Mississippi State University Institutional Animal Care and Use Committee and conformed with the Guide for the Care and Use of Laboratory Animals published by the US National Institutes of Health (NIH publication no. 85–23, revised 2011).

### 2.2. Echocardiography

Adult mice (4–6 months old) were lightly anaesthetized using 1–1.5% isoflurane. In vivo cardiac function was assessed using a Vevo 3100 ultrasound machine (Visual Sonics, Toronto, ON, Canada). Data were analyzed using Visual Sonics software version 3.3.1 (Visual Sonics). Data presented in Table 1 were analyzed from the M-mode images.

### 2.3. Cardiomyocyte Isolation

Ventricular myocytes were isolated from mice hearts. After the mice were fully anesthetized [22], hearts were quickly excised, attached to a Langendorff apparatus via cannulating needle, and perfused with 37 °C Tyrode solution (140 mM NaCl, 10 mM HEPES, 5.6 mM Glucose, 5.4 mM KCl, 0.5 mM MgCl_2_, pH 7.30 with NaOH) for 3 min. Next, hearts were perfused with Tyrode solution containing Liberase TH (Roche, Basel, Switzerland) for 10–15 min to digest connective tissue. Ventricular cardiomyocytes were then isolated from the digested ventricular tissue and stabilized in Tyrode containing bovine serum albumin (BSA). After being gradually reintroduced to Ca^2+^, cells were plated on laminin-coated coverslips to be used in cellular imaging experiments.

### 2.4. Fluorescent Imaging

Fluorescence was monitored using an inverted epifluorescent microscope (Olympus IX83, Breinigsville, PA, USA) and recorded using a Hamamatsu ORCA-Flash4.0 V3 Digital CMOS camera (maximum speed: 100 frames/s).

#### 2.4.1. Ca^2+^ Imaging

Ventricular myocytes were loaded with Ca^2+^-sensitive dye Fluo-3 AM (~8 µM in 1 mM Ca^2+^ Tyrode) for 25 min at room temperature, followed by a wash solution of 1 mM Ca^2+^ Tyrode for 25 min to allow for de-esterification. To measure cytosolic Ca^2+^ dynamics, fluo-3 was excited at 480 ± 20 nm and emissions were measured at 535 ± 25 nm. Cells were recorded under baseline conditions (perfusing with 1 mM Ca^2+^ Tyrode) or with β-adrenergic stimulation (perfusing with 1 mM Ca^2+^ Tyrode solution containing 100 nM isoproterenol, ISO). Cells were paced with a platinum electrode using field stimulation at 0.5 Hz to measure the properties of the Ca^2+^ transient and propensity to generate diastolic Ca^2+^ waves.

X-Rhod-1/AM (excited at 560 ± 20 nm, emissions measured at 630 ± 25 nm) was utilized to measure mitochondrial Ca^2+^ [23,24]. Myocytes were loaded with the dye (~5 µM in 1 mM Ca^2+^ Tyrode) for 30 min at 37 °C. Next, the cytosolic Ca^2+^ signal was quenched with 1 mM Ca^2+^ Tyrode containing 1 mM CoCl_2_ for 25 min. Before recordings were taken, cells were paced to ensure no cytosolic Ca^2+^ transient was observed. Cells were paced at 1 Hz and perfused with 100 nM ISO to induce mitochondrial Ca^2+^ accumulation.

#### 2.4.2. ROS Imaging

Cellular ROS generation was measured using the fluorescent indicator CM-H2DCFDA (2 µM). Cells were loaded for 25 min at room temperature, followed by a wash solution of 1 mM Ca^2+^ Tyrode for 25 min. CM-H2DCFDA was excited at 480 ± 20 nm and emissions were measured at 535 ± 25 nm. Cardiomyocytes were perfused with 1 mM Ca^2+^ Tyrode (with or without 100 nM ISO) and paced at 0.5 Hz during the recording. Fluorescence was normalized to the maximum signal obtained with the application of 8.82 mM H_2_O_2_ at the end of the experiment. Twenty-one images were taken during a time window of 5 min before the addition of H_2_O_2_. The rate of ROS production during perfusion was quantified by fitting the data with the ordinary least-squares regression line.

#### 2.4.3. Mitochondrial Membrane Potential (MitoWinks Recordings)

The brief opening of mPTP or MitoWinks was recorded using the voltage-sensitive dye TMRE (50 nM) [18]. Cells were loaded with TMRE in 1 mM Ca^2+^ Tyrode for 15 min to measure mitochondrial membrane potential. TMRE was excited at 560 ± 20 nm, and emissions were measured at 630 ± 25 nm. During the recording, cells were perfused in 1 mM Ca^2+^ Tyrode containing 100 nM ISO for 20 min. MitoWinks were detected as a brief decrease and recovery in fluorescence in individual mitochondria. The number of MitoWinks was quantified and used in statistical analysis.

### 2.5. Western Blot

Proteins were extracted from cardiac tissue after homogenization in an extraction buffer supplemented with protease and phosphatase inhibitors. Proteins were separated using SDS-PAGE and transferred onto nitrocellulose membranes. The membranes were probed using primary antibodies against CaMKII, CaMKII (Phospho-Thr286), RyR2, RyR2 (Phosph-Ser2814), and GAPDH followed by secondary horseradish peroxidase-conjugated antibodies. SuperSignal chemiluminescence (Pierce Biotechnology Inc, Rockford, IL, USA) was utilized to detect the protein bands. ImageJ was utilized to quantify the protein expression levels.

### 2.6. Staining of Cardiac Tissue Sections

Mouse hearts were fixed in 10% phosphate-buffered formaldehyde (pH 7.4), paraffin embedded, and sectioned longitudinally at 5 μm thickness. Cardiac tissue sections were stained with hematoxylin and eosin (H&E) to detect changes in the morphology of the heart. Wheat germ agglutinin (WGA) staining was performed to assess cardiomyocyte cross-sectional area. Tissue sections were stained with WGA (1:100 dilution) following deparaffinization and antigen retrieval. After staining, slides were imaged with an Olympus IX83 inverted fluorescent microscope. Cross-sectional areas of around 65 myocytes per mouse heart were analyzed using ImageJ software to determine cellular hypertrophy.

### 2.7. RNA Extraction, Library Construction, and Sequencing

RNA was extracted from freshly harvested mouse heart tissue with RNAzol Reagent, following the manufacturer’s protocol (Molecular Research Center, Inc. Cincinnati, OH, USA). At least 3 mice from each experimental group were utilized for RNA extraction. Libraries were constructed following an established protocol [25]. All the libraries were analyzed and quantified with a bioanalyzer and sequenced on an Illumina NEXTSeq 2000 system using a fee-based service at the Weill Cornell Genomic Core Facility. All sequence data of the 13 libraries were deposited in the NCBI Sequence Read Archive (SRA) database under the accession number PRJNA916203.

### 2.8. mRNA Sequence Processing

The raw reads were checked using FastQC (http://www.bioinformatics.babraham.ac.uk/projects/fastqc/, accessed on 15 July 2022) to determine the sequencing quality. After that, the sequencing adapters and low-quality bases were removed from raw reads using trimmomatic [26] and the trimmed raw reads shorter than 75 nt were also discarded. The remaining clean reads were aligned to the mouse reference genome (*Mus Musculus* GRCm39, https://asia.ensembl.org/Mus_musculus/Info/Index, accessed on 26 October 2021) sequence using the STAR program allowing up to two mismatches [27]. The number of reads mapping to genes was counted using featureCounts [28], then the raw counts were normalized into FPKM (fragments per kilobase of exon model per million mapped fragments).

Differential expression analysis was performed using the DESeq2 R package Version 1.30.1 [29]. The *p*-values were adjusted using the Benjamini and Hochberg method. A corrected *p*-value of 0.05 and log2FC (fold change) of 1 were set as the thresholds to determine the significantly changed genes. Gene ontology (GO) enrichment analysis of differentially expressed genes was performed with the clusterProfiler 4.0 R package [30]. GO terms with *p*-values less than 0.05 were considered significantly enriched.

### 2.9. Statistical Analysis

Data were compared using an unpaired Student’s *t*-test or one-way ANOVA test when appropriate; all values were reported as means ± standard error of the mean (SEM), unless otherwise noted. A *p*-value < 0.05 was considered statistically significant.

## 3. Results

### 3.1. DKO Mice Did Not Display Pathological Cardiac Remodeling

**Figure 1:**
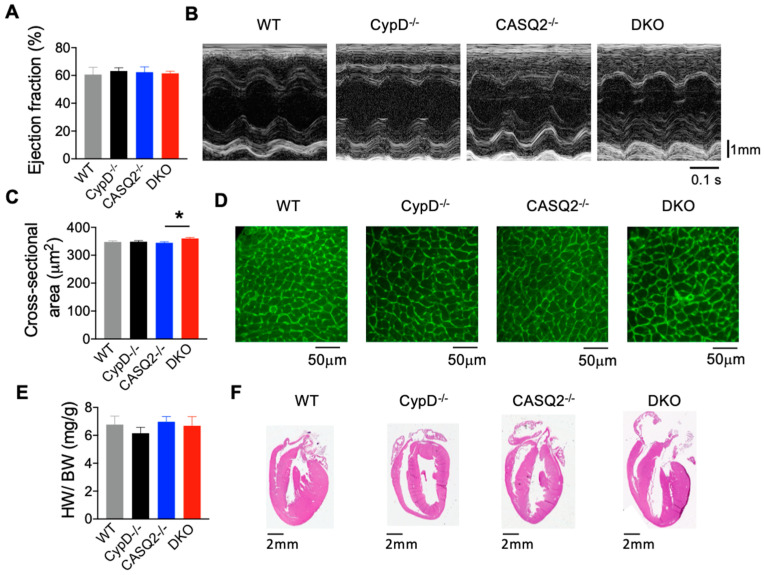
Deletion of CypD in CASQ2^−/−^ mice did not cause pathological cardiac remodeling. (**A**) Mean ± SEM of left ventricular EF in CypD^−/−^ (n = 9), CASQ2^−/−^ (n = 8), DKO (n = 8), and WT (n = 6) mice. (**B**) Representative images of M-mode echocardiography of left ventricle in the different groups. (**C**) Mean ± SEM of cross-sectional area of myocytes as measured with WGA staining in WT (n = 195 cells, N = 3 hearts), CypD^−/−^ (n = 195 cells, N = 3 hearts), CASQ2^−/−^ (n = 195 cells, N = 3 hearts), and DKO (n = 195 cells, N = 3 hearts). * *p* < 0.05 one-way ANOVA between groups. (**D**) Representative images of the WGA staining. (**E**) Mean ± SEM of heart weight/body weight (HW/BW) ratio in WT (n = 7), CypD^−/−^ (n = 8), CASQ2^−/−^ (n = 4) and DKO (n = 6) mice. (**F**) Representative images of H&E staining of the hearts from different groups.

The knockout of both CASQ2 and CypD did not cause premature death of the animals. We characterized the in vivo cardiac function of the DKO mice (5 months old) using echocardiography. We did not detect significant differences in the cardiac ejection fraction (EF) or fractional shortening between the DKO mice, the two single KO models, and the WT control (Figure 1A,B; Table 1). We also analyzed other parameters and found no difference in the E/A-wave ratio, interventricular septal end diastole and systole (IVS;d and IVS;s), or left ventricular internal diameter end diastole and systole (LVID;d and LVID;s). These results suggest that DKO mice had nearly normal cardiac contractility and unimpaired relaxation. The unaltered chamber dimensions suggest that pathological remodeling, such as dilation, did not occur. We found that the thickness of the left ventricular posterior wall diastole and systole (LVPW;d and LVPW;s) were both increased in DKO as compared with WT (Table 1); however, the heart weight to body weight ratio for DKO was not different from that of WT and the two single KO controls (Figure 1E). Additionally, H&E staining of cardiac tissue sections did not reveal structural remodeling of the heart (Figure 1F). When we quantified the cross-sectional area of myocytes using WGA staining, the DKO cells had a slight but significant increase in cross-sectional area as compared with the CASQ2^−/−^ (Figure 1C,D). However, there was no difference between the DKO and WT control. Overall, these results suggest that the genetic inhibition of mPTP in the CPVT model of CASQ2 KO did not lead to overt pathological cardiac remodeling, despite some early signs of potential cardiac hypertrophy.

**Table 1:** Echocardiography parameters in the different experimental groups.

Echocardiography Parameters	CypD^−/−^	CASQ2^−/−^	DKO	WT
E/A	1.60 ± 0.12	1.76 ± 0.18	1.34 ± 0.05	1.37 ± 0.13
IVS;d mm	1.12 ± 0.10	1.18 ± 0.07	1.44 ± 0.16	1.03 ± 0.06
IVS;s mm	1.57 ± 0.12	1.64 ± 0.09	1.76 ± 0.12	1.53 ± 0.10
LVID;d mm	3.17 ± 0.14	3.46 ± 0.14	3.05 ± 0.10	3.50 ± 0.15
LVID;s mm	2.19 ± 0.11	2.30 ± 0.10	2.21 ± 0.39	2.38 ± 0.21
LVPW;d mm	1.31 ± 0.10 *	1.09 ± 0.10	1.38 ± 0.10 *	0.83 ± 0.10
LVPW;s mm	1.52 ± 0.14	1.49 ± 0.11	1.63 ± 0.09 *	1.10 ± 0.12
LV Vol;s µL	16.50 ± 2.21	18.43 ± 1.98	17.19 ± 3.10	24.29 ± 2.08
LV Vol;d µL	41.11 ± 4.25	50.33 ± 4.95	36.93 ± 2.92	51.41 ± 5.05
Ejection Fraction %	63.15 ± 2.43	62.38 ± 3.76	61.48 ± 1.63	60.66 ± 5.18
Fractional Shortening %	33.40 ± 1.72	33.31 ± 2.65	32.02 ± 2.03	32.46 ± 4.05
Heart Rate	446 ± 31	400 ± 20	424 ± 20	431 ± 19

The value represents the mean ± SEM, n = 6–9 mice per group. * *p* < 0.05 vs. WT, analyzed by one way ANOVA. E/A-wave ratio indicates E-wave and A-wave that represent the left ventricular early filling and atrial contraction filling, respectively; IVS;d, interventricular septal end diastole; IVS;s, interventricular septal end systole; LVID;d, left ventricular internal diameter end diastole; LVID;s, left ventricular internal diameter end systole; LVPW;d, left ventricular posterior wall end diastole; LVPW;s, left ventricular posterior wall end systole.

### 3.2. DKO Mice Displayed Abnormal Myocyte Ca^2+^ Handling

Intracellular Ca^2+^ dynamics were examined in WT, CypD^−/−^, CASQ2^−/−^, and DKO myocytes using fluorescent microscopy and Fluo-3. The parameters of Ca^2+^ transients and spontaneous Ca^2+^ waves (SCWs) were measured in field-stimulated myocytes under baseline conditions and after perfusion with the β-agonist, ISO. Cells were paced at 0.5 Hz continuously so that we could assess the property of both systolic Ca^2+^ transients and diastolic Ca^2+^ waves simultaneously.

**Figure 2:**
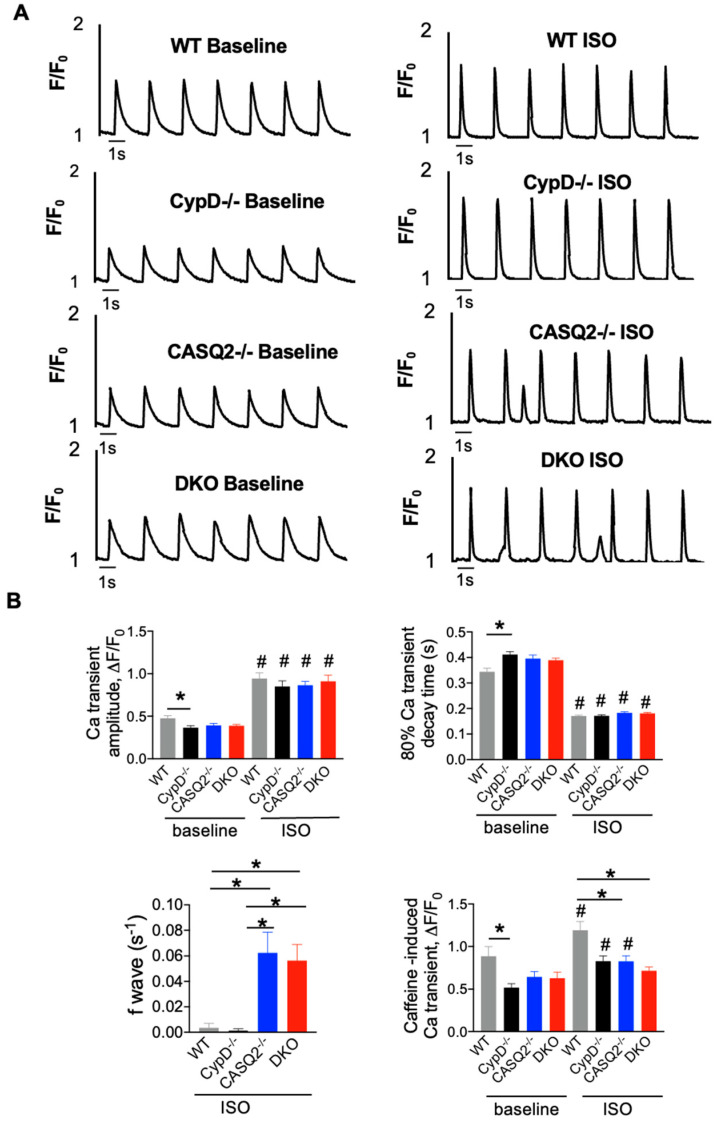
Intracellular Ca^2+^ handling of myocytes isolated from WT, CypD^−/−^, CASQ2^−/−^, and DKO hearts. (**A**) Representative traces of time-dependent fluorescence profiles of Ca^2+^ transient and SCWs under baseline condition and in the presence of 100 nM ISO. (**B**) Mean ± SEM of average Fluo-3 amplitude (ΔF/F_0_) (n = 28–70 cells from N = 3–4 mice), 80% Ca^2+^ transient decay time (n = 28–70 cells from N = 3–4 mice), frequency of Ca^2+^ waves (n = 36–82 cells from N = 3–4 mice), and SR Ca^2+^ content (ΔF/F_0_) (n = 21–42 cells from N = 3–4 mice). * *p* < 0.05 one-way ANOVA between groups, # *p* < 0.05 vs. baseline.

When paced at 0.5 Hz, CypD^−/−^ myocytes had a lower Ca^2+^ transient amplitude and a slower Ca^2+^ transient decay kinetics when compared with WT control (Figure 2A,B). CASQ2^−/−^ and DKO had similar Ca^2+^ transient amplitude and Ca^2+^ transient decay kinetics, both of which were not different from that of the WT control (Figure 2A,B). ISO significantly increased the Ca^2+^ transient amplitude and kinetics of Ca^2+^ transient decay in all four groups (Figure 2A,B). We did not detect a significant difference in the Ca^2+^ transient amplitude or the kinetics of Ca^2+^ transient decay among the four groups with ISO perfusion. The frequency of SCWs was quantified to evaluate the cellular arrhythmic burden. Similar to the WT control, the CypD^−/−^ rarely displayed any SCWs (Figure 2A,B). In contrast, the frequency of SCWs was found to be significantly higher in CASQ2^−/−^ than in WT and CypD^−/−^, which was consistent with the proarrhythmic phenotype of the CPVT model [20]. However, DKO myocytes had a similar frequency of waves to CASQ2^−/−^. The SR Ca^2+^ content was estimated by measuring the amplitude of caffeine-induced Ca^2+^ transient. The SR Ca^2+^ content was found to be similar across the three mutant groups under baseline conditions. CypD^−/−^ had the lowest SR Ca^2+^ content, which was significantly lower than that of the WT control, which may explain its lower Ca^2+^ transient amplitude. ISO increased the SR Ca^2+^ content in the two single KO groups and WT control, but did not further increase the SR Ca^2+^ content in DKO myocytes (Figure 2B). Both CASQ2^−/−^ and DKO had a significantly lower SR Ca^2+^ content than the WT control, which was explained by the frequent development of SCWs, which reduced the SR Ca^2+^ load.

### 3.3. Genetic Inhibition of mPTP in the CPVT Model Led to CaMKII-Mediated Hyperphosphorylation of RyR2

**Figure 3:**
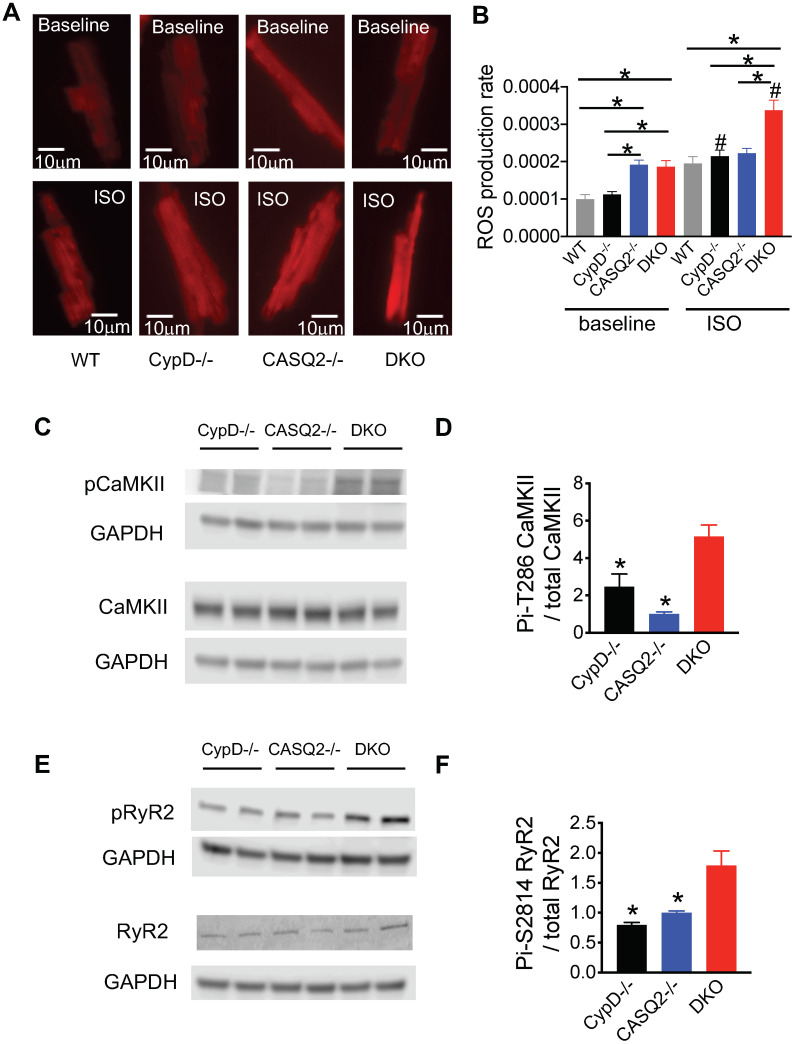
Increased autonomous activation of CaMKII led to higher levels of Ser-2814 phosphorylation of RyR2 in DKO hearts. (**A**) Representative images of cellular ROS production in WT, CypD^−/−^, CASQ2^−/−^, and DKO. For each recording, 21 images were captured during a 5 min time window. The last image at the 5 min time point was shown as the representative. (**B**) Mean ± SEM of average ROS production rate in the three groups. N = 18–51 cells from N = 3–4 mice, * *p* < 0.05 one way-ANOVA between groups, # *p* < 0.05 vs. baseline. (**C**) Representative western blots detecting phosphorylation of CaMKII at Thr-286 and its expression in CypD^−/−^, CASQ2^−/−^, and DKO; GAPDH served as loading control. (**D**) Mean ± SEM, quantification of western blots; * *p* < 0.05 compared with DKO, analyzed by one-way ANOVA, N = 4 hearts for each group. (**E**) Representative western blots detecting phosphorylation of RyR2 at Ser-2814 and its expression in CypD^−/−^, CASQ2^−/−^, and DKO; GAPDH served as loading control. (**F**) Mean ± SEM, quantification of western blots; * *p* < 0.05 compared with DKO, analyzed by one-way ANOVA, N = 4 hearts for each group.

Our previous study provided evidence that the inhibition of mPTP in the setting of CPVT exacerbated RyR2 dysfunction [19]. We found that treating CPVT cells with a pharmacological inhibitor of mPTP, CsA, exacerbated the Ca^2+^ waves in the CASQ2^−/−^ myocytes [19]. The genetic inhibition of mPTP in the CASQ2^−/−^ model also led to more severe ventricular arrhythmias [19]. We hypothesize that the exacerbation of RyR2 dysfunction was due to excessive ROS emission, activation of the CaMKII pathway, and hyperphosphorylation of RyR2. Our previous results indicated that both the pharmacological and genetic inhibition of mPTP increased mitochondrial ROS production [19]. We further evaluated cellular ROS production using a generic ROS indicator CM-H2DCFDA in this study (Figure 3A,B). Under baseline conditions, CypD^−/−^ cells had a normal ROS production rate, similar to that of the WT control. DKO and CASQ2^−/−^ cells had a similar level of ROS production rate, which was significantly higher than that of WT or CypD^−/−^ cells (Figure 3A,B). With ISO perfusion, CypD^−/−^ and CASQ2^−/−^ had similar levels of ROS production to that of the WT cells. However, DKO cells had a much higher ROS production rate than WT, CypD^−/−^, or CASQ2^−/−^ cells (Figure 3A,B). Next, we examined the activation of the CaMKII pathway by measuring its autophosphorylation at Thr-286. We detected a higher level of phosphorylation at Thr-286 of CaMKII in the DKO cardiac lysate than in the control groups (Figure 3C,D). Further, the phosphorylation of RyR2 at Ser-2814, the CaMKII site, was also significantly higher in the DKO cardiac lysate than in the control groups (Figure 3E,F). Collectively, these results suggest that the genetic inhibition of mPTP in the CPVT model led to higher ROS production, activation of the CaMKII pathway, and subsequent hyperphosphorylation of RyR2 in the DKO model, thus, further exacerbating RyR2 dysfunction. Indeed, when the DKO cells were pretreated with the CaMKII inhibitor KN93, the frequency of arrhythmogenic Ca^2+^ waves was significantly reduced (Supplemental Figure S1), thus, supporting the functional relevance of the activation of the CaMKII pathway in RyR2 dysfunction.

### 3.4. Transcriptome Analysis Identified Altered Gene Expression Patterns Associated with Electrical Instability in DKO

**Figure 4:**
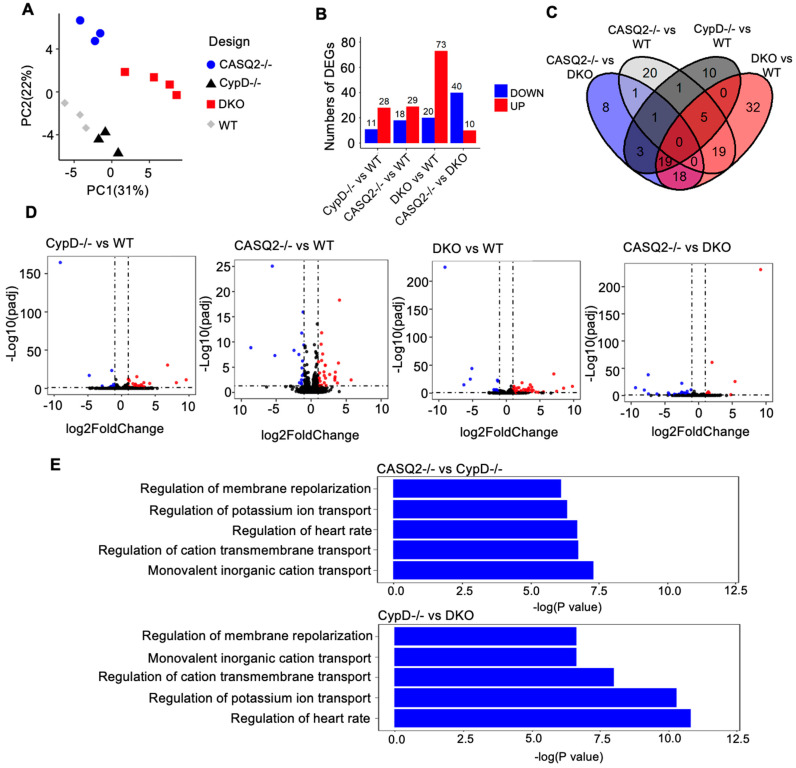
Transcriptome analysis identified altered gene expression patterns associated with electrical instability in DKO. (**A**) PCA plot showing the distinct gene expression between groups. (**B**) Number of differentially expressed genes when compared between the different genotypes. Number of significant upregulated genes (red) based on log2FC > 1&p_adj_ < 0.05, and number of significant down-regulated genes (blue) based on log2FC < −1&p_adj_ < 0.05. (**C**) Venn diagrams showing the intersection between significantly regulated genes (p_adj_ < 0.05 & log2FC > 1). (**D**) Volcano plot of all genes in different comparisons. Significant upregulated genes (red) based on log2FC > 1 and p_adj_ < 0.05, and significant downregulated genes (blue) based on log2FC < −1 and p_adj_ < 0.05. (**E**) GO term enrichment analysis of differentially expressed genes.

RNAseq was performed with RNA extracted from WT, CypD^−/−^, CASQ2^−/−^, and DKO hearts to obtain a global overview of transcriptional differences between the genotypes. The initial quality control of principal component analysis (PCA) demonstrated segregation of the four groups (Figure 4A). We first compared each of the mutant groups with the WT control to identify differentially expressed genes (DEGs). We found 39 DEGs (28 upregulated and 11 downregulated) in CypD^−/−^, 47 DEGs (29 upregulated and 18 downregulated) in CASQ2^−/−^, and 93 DEGs (73 upregulated and 20 downregulated) in DKO (Figure 4B). The highest number of DEGs found in DKO suggests that this group had more extensive transcriptome remodeling. To better understand the pathways altered by genetic manipulations, we performed pathway enrichment analysis (Supplemental Table S1). When comparing WT with CASQ2^−/−^ or DKO, the DEGs were enriched in pathways controlling ion transport and, thus, regulating the heart rhythm. These pathways include the regulation of cation transmembrane transport, regulation of potassium ion transmembrane transport, regulation of heart rate, and the regulation of membrane repolarization (Supplemental Table S1). In contrast, none of these pathways were enriched when comparing WT to CypD^−/−^. Indeed, while both CASQ2^−/−^ and DKO displayed cellular and in vivo arrhythmias, CypD^−/−^ is not known to exhibit a proarrhythmic phenotype and did not show arrhythmogenic Ca^2+^ waves in our cellular assays (Figure 2). The DEGs involved in the heart rhythm-related pathways were summarized in Supplemental Figure S2.

We also looked into the expression of genes that are involved in mCa^2+^ homeostasis, including MCU and its several modulatory proteins, as well as RyR1, mNCX, and Letm1. We did not detect any changes in the expression of these genes (Supplemental Figure S3). Several ER/SR localized channels, such as inositol 1,4,5-trisphosphate (IP3) receptors [31] and trimeric intracellular cation (TRIC) channels [32,33], have been shown to crosstalk with RyR2 to impact intracellular Ca^2+^ signaling. In addition, store-operated Ca^2+^ entry (SOCE) has recently emerged as a pathway to regulate myocyte Ca^2+^ homeostasis and has been implicated in cardiac pathologies including arrhythmias [34]. However, we did not detect differences in the expression of these channels or proteins involved in SOCE pathways (Supplemental Figure S4).

We further compared the mutant groups (Supplemental Table S2). Similar to when compared with WT, the DEGs in CASQ2^−/−^ and DKO were enriched in pathways involved in the ion transport and regulation of heart rhythm when compared with CypD^−/−^(Supplemental Table S2). Importantly, the changes in these pathways were more significant between CypD^−/−^ and DKO than CypD^−/−^ and CASQ2^−/−^, as demonstrated by the much lower *p*-values (Figure 4E). This was consistent with the more severe arrhythmic phenotype of the DKO mice [19]. We also identified some genes that were only altered in the DKO, but were expressed at similar levels between the three control groups. The majority of these genes have not been linked to cardiac pathology before, and some of them are predicted genes with unknown functions (Supplemental Figure S5). Interestingly, Xist, a non-coding RNA upregulated in several heart diseases [35,36], was drastically upregulated in DKO, but remained low in WT, CASQ2^−/−^, and CypD^−/−^. Nevertheless, it remains unknown if and how these genes may contribute to cardiac pathologies, and this requires further study.

## 4. Discussion

**Figure 5:**
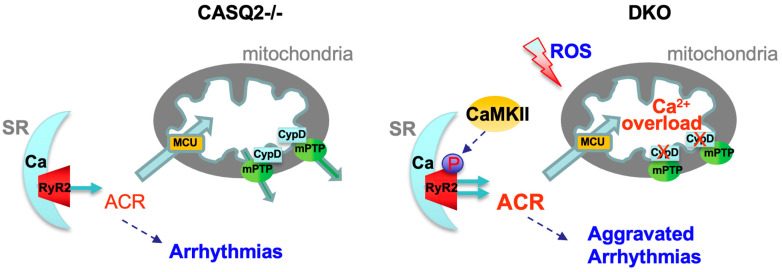
Genetic inhibition of mPTP exacerbates RyR2 dysfunction in CPVT by increasing the autonomous activation of CaMKII and subsequent hyperphosphorylation of RyR2.

In this study, we generated a DKO model missing both CypD and CASQ2, and investigated the consequence of genetic inhibition of mPTP in the CPVT model of CASQ2^−/−^ mouse. The DKO model exhibited a normal cardiac contractility and HW/BW ratio and did not display overt cardiac remodeling. Although the DKO model displayed frequent arrhythmogenic Ca^2+^ waves when paced at a low frequency (0.5 Hz), myocyte Ca^2+^ handling was not aggravated when compared with the CASQ2^−/−^ model. Interestingly, our previous study found that when increasing the pacing frequency to 2 Hz, both Ca^2+^ transient amplitude and SR Ca^2+^ content were depressed in the DKO myocytes compared with the CASQ2^−/−^ [19]. We also reported that the DKO mice developed more severe ventricular arrhythmias than the CASQ2^−/−^ mice, thus, suggesting aggravated RyR2 dysfunction [19]. In further exploring the molecular mechanism underlying the exacerbation of RyR2 dysfunction, we discovered elevated cellular oxidative stress levels and activation of the CaMKII signaling pathway in the DKO mice, resulting in hyperphosphorylation of RyR2 (Figure 5). Additionally, transcriptome analysis identified a gene expression signature marked by increased electrical instability in the DKO mice.

### 4.1. Frequency-Dependent Exacerbation of Ca^2+^ Handling

Previously, we found that, when paced at 2 Hz, DKO myocytes presented exacerbated myocyte Ca^2+^ handling compared with CASQ2^−/−^ myocytes [19]. Interestingly, when paced at a lower frequency of 0.5 Hz, DKO myocytes did not show aggravation in Ca^2+^ handling as compared with CASQ2^−/−^. They had similar Ca^2+^ transient amplitude, kinetics of Ca^2+^ transient decay, as well as frequency of arrhythmogenic Ca^2+^ waves. This was consistent with the rather mild cardiac pathological phenotype of the DKO mice. Besides the exacerbation of ventricular arrhythmias, we did not observe structural cardiac remodeling. It is known that during a fight or flight response, more Ca^2+^ enters the mitochondria to boost energy production to match the increased metabolic demand [5]. When perfused with the β-agonist ISO, systolic Ca^2+^ transient amplitude increases and, thus, enhances Ca^2+^ transfer to the mitochondria. Moreover, in the setting of CPVT, ISO also elevates RyR2-mediated ACR, thus, increasing the mCa uptake during diastole. Increasing the pacing frequency is expected to load mitochondria with more Ca^2+^ due to more frequent systolic Ca^2+^ transients, as well as elevated diastolic ACR, which is synchronized by pacing [37]. Furthermore, high-frequency pacing may cause the autonomous activation of CaMKII [38] to phosphorylate RyR2 to compound the defects of the channel complex, as discussed further below. Thus, with an increased pacing frequency, CPVT myocytes are more susceptible to mCa^2+^ overload, and the aggravation in myocyte Ca^2+^ handling due to mPTP inhibition is more likely to be revealed.

### 4.2. Role of CaMKII in CPVT

CPVT is a stress-induced arrhythmia syndrome. Following β-adrenergic stimulation and subsequent activation of downstream kinases, several important targets, including the L-type Ca^2+^ channel, phospholamban, Troponin I, and RyR2, are phosphorylated in cardiomyocytes, contributing to the positive inotropy and lusitropy of the fight or flight response [39]. At the same time, enhanced Ca^2+^ handling increases the SR Ca^2+^ load to precipitate RyR2 leak and the generation of arrhythmogenic Ca^2+^ waves. While the role of PKA in the β-adrenergic pathway is well established, emerging evidence has also suggested a critical role of CaMKII [40,41]. RyR2 can be phosphorylated by both PKA and CaMKII at multiple sites. The role of its phosphorylation at Ser-2808 by PKA in the fight or flight response or cardiac pathologies remains highly controversial [42,43,44,45]. In contrast, studies from different labs have provided evidence that CaMKII phosphorylation of RyR2 at Ser-2814 exacerbates RyR2 leak and contributes to multiple cardiac dysfunctions including heart failure and arrhythmias [46,47,48]. In particular, a recent study showed that RyR2 phosphorylation at Ser-2814 by CaMKII is required to unmask the arrhythmic potential of engineered human heart tissue of CPVT [49]. Moreover, CaMKII inhibition prevents ventricular arrhythmias in mouse models of CPVT, and induced pluripotent stem cell-derived cardiomyocytes of CPVT patients [50]. It is likely CaMKII phosphorylation of RyR2 further aggravates the defects of the channel complex caused by CPVT mutations, thus, contributing to cardiac arrhythmias.

The initial activation of CaMKII relies on elevated Ca^2+^ levels. Ca^2+^-bound calmodulin binds to the regulatory domain of CaMKII and blocks the association between its regulatory and catalytic domains, thus, relieving the autoinhibition of the catalytic domain and resulting in the direct activation of the enzyme [38]. The binding of Ca^2+^/CaM to CaMKII is required as a first step for all known mechanisms that activate CaMKII. Prolonged Ca^2+^/CaM binding to CaMKII—as observed during high frequency Ca^2+^ transient—leads to its autophosphorylation at Thr-286, resulting in the autonomous activation of the enzyme even after the dissociation of Ca^2+^/CaM [38]. Recent studies have also demonstrated the ROS-dependent autonomous activation of CaMKII [51] and its implication in numerous cardiac pathological processes [38,52]. We hypothesize that the genetic inhibition of mPTP-mediated Ca^2+^ efflux leads to mCa^2+^ overload-dependent ROS production, which leads to a further activation of CaMKII and hyperphosphorylation of RyR2 at Ser 2814 to exacerbate RyR2 leak and arrhythmias. Indeed, we detected higher oxidative stress in the DKO myocytes, as well as increased activation of the CaMKII pathway and RyR2 phosphorylation at Ser-2814.

### 4.3. The Detrimental Effect of mPTP Inhibition in Cardiac Dysfunctions Marked by Leaky RyR2

It has been shown that the inhibition of mPTP (genetic or pharmacological approaches) reduces cardiac damage in ischemia-reperfusion injury [11,12,13,14,15,16]. However, recent studies also provided evidence supporting the physiological function of mPTP-mediated Ca^2+^ efflux, which acts as a Ca^2+^ release valve to avoid mCa^2+^ overload [18]. Thus, it is important to evaluate the potential harmful consequence of mPTP inhibition, especially in disease settings that predispose mitochondria to Ca^2+^ overload. One might expect that, in disease conditions associated with RyR2 dysfunction (i.e., RyR2 leak), mitochondria may rely more heavily on mPTP-mediated Ca^2+^ efflux to avoid mCa^2+^ overload as SR-derived leaked Ca^2+^ may travel to the mitochondria. Indeed, we observed that the frequency of MitoWinks increased significantly in CPVT and another disease model also marked by RyR2 dysfunction in our previous study [19]. Thus, we hypothesize that the inhibition of mPTP in CPVT is likely to result in detrimental consequences. This was supported by our previous study, which demonstrated that cardiac arrhythmias were exacerbated in the CPVT model of CASQ2^−/−^ when mPTP was inhibited. Furthermore, consistent with our study, the genetic inhibition of mPTP opening exacerbated cardiac pathologies in mice subjected to a pressure overload or overexpression of CaMKII, both of which feature RyR2 dysfunction (leak) [53]. When subjected to pressure overload, the CypD^−/−^ mice exhibited more severe hypertrophy, fibrosis, and deterioration in cardiac contractility [53]. The overexpression of CaMKII in CypD^−/−^ exacerbated premature death and pathological cardiac remodeling. CypD^−/−^ mice also displayed increased mCa^2+^ [53]. Collectively, these studies suggest that blocking mPTP-mediated mCa^2+^ efflux may lead to detrimental effects due to mCa^2+^ overload and, thus, therapies targeting mPTP should be designed with caution. Notably, the current study was conducted with mouse models. Mouse models of CPVT (CASQ2 KO [20] and several other lines [54,55,56]) have been reported to recapitulate the human disease and have been extensively used to facilitate mechanistic studies of CPVT and RyR2 dysfunction [37,57,58,59,60]. However, considering the species-dependent differences, results obtained from here or mouse studies in general need to be cautiously evaluated or verified in large animal models before clinical application.

### 4.4. Electrical Remodeling Identified with Transcriptome Analysis

Our RNAseq data revealed altered gene expression between the DKO and control groups. When compared with WT, DEGs were identified in all three mutant groups. For both CASQ2^−/−^ and DKO, which displayed cellular and in vivo arrhythmias [19], DEGs were enriched in pathways involved in ion transport or regulation, thus, impacting heart rhythm. In contrast, these pathways were not impacted in CypD^−/−^, which is not an arrhythmic model. We also compared the expression of a few genes involved in maintaining mCa^2+^ homeostasis and intracellular Ca^2+^ signaling. We did not find any significant changes in the genes regulating mCa^2+^ uptake and extrusion in any model compared to WT (Supplemental Figure S3). We also found no difference in the expression of IP3R, TRIC, and several key proteins involved in the SOCE pathway (Supplemental Figure S4), all of which may crosstalk with RyR2 to impact Ca^2+^ signaling. It is still possible these channels or proteins affect RyR2 functionality or Ca^2+^ signaling in the DKO model through post-translational modification or altered interprotein interactions, but this requires further study. Additionally, we identified some genes in DKO that had a unique expression pattern compared to the three control groups. The functional relevance of most of these genes in cardiac pathology remains unknown and requires future studies.

### 4.5. Limitations

Both the prolonged and brief opening modes of mPTP are inhibited in the CypD^−/−^ model [9,18,61,62]. The prolonged opening of mPTP is usually associated with the activation of cell death pathways. Since the CPVT model was marked by electrical instability but did not exhibit pathological cardiac remodeling, the prolonged opening mode may have been less relevant than the brief opening mode in this setting. Still, we cannot exclude the possibility that the observed phenotype of DKO may be due to the inhibition of both opening modes of mPTP. Nevertheless, we did observe that MitoWinks, an indicator of the brief opening mode or mPTP-mediated Ca^2+^ efflux, was significantly inhibited in the DKO cells compared to the CASQ2^−/−^ cells (Supplemental Figure S6). Moreover, when measuring mCa^2+^ with X-Rhod-1 in intact cells, we found that pacing-induced mCa^2+^ accumulation (mCa^2+^ peak) was higher in DKO cells than CASQ2^−/−^ cells, suggesting an mCa^2+^ overload in DKO (Supplemental Figure S7). Together, these results supported our hypothesis that the inhibition of the brief opening of mPTP (mPTP-mediated Ca^2+^ efflux) in DKO leads to mCa^2+^ overload, thus, exacerbating ROS production, and eventually contributing to the aggravation of arrhythmias.

## 5. Conclusions

In summary, our study provides evidence that the genetic inhibition of mPTP exacerbates RyR2 dysfunction in CPVT by increasing the autonomous activation of CaMKII and subsequent hyperphosphorylation of RyR2. A transcriptome analysis of the novel DKO model revealed a gene expression profile consistent with the pathological phenotype of the model.

## Data Availability

All sequence data will be deposited in the NCBI Sequence Read Archive (SRA) database after the publication of the manuscript.

## Supplementary Materials

The following supporting information can be downloaded at: https://www.mdpi.com/article/10.3390/cells12020204/s1, Table S1: Differentially expressed genes and GO term enrichment analysis (Compared with WT). Table S2: Differentially expressed genes and GO term enrichment analysis (Compared between mutant groups). Figure S1: The CaMKII inhibitor KN93 reduced the frequency of Ca^2+^ waves in the DKO cells. Figure S2: RPKM (reads per kilobase of exon per million reads mapped) levels of genes involved in pathways regulating ion transport and thus impacting heart rhythm. Figure S3: RPKM levels of genes involved in mCa^2+^ handling. Figure S4: RPKM levels of IP3R, TRIC channels and proteins in the SOCE pathway. Figure S5: RPKM levels of transcripts that were expressed differentially in the DKO group. Figure S6: the transient opening mode of mPTP was inhibited in the DKO cells. Figure S7: Increased mitochondrial Ca^2+^ in DKO.

## Abbreviations

ACR	Aberrant calcium release
BSA	Bovine Serum Albumin
Ca^2+^	Calcium
CPVT	Catecholaminergic polymorphic ventricular tachycardia
DEGs	Differentially expressed genes
DKO	Double Knockout
E/A-wave ratio	E-wave and A-wave that represent the left ventricular early Filling and atrial contraction filling, respectively
EF	Ejection fraction
FC	Fold Change
GO	Gene Ontology
H&E	Hematoxylin and Eosin
HW/BW	Heart weight/body weight
ISO	Isoproterenol
IVS;d	Interventricular septal end diastole
IVS;s	Interventricular septal end systole
KO	Knockout
LVID;d	Left ventricular internal diameter end diastole
LVID;s	Left ventricular internal diameter end systole
LVPW;d	Left ventricular posterior wall end diastole
LVPW;s	Left ventricular posterior wall end systole
mCa^2+^	Mitochondrial Calcium
MCU	Mitochondrial calcium uniporter
MitoWinks	Transient opening of mPTP
mNCX	Mitochondrial Na/Ca (sodium/calcium) exchanger
mPTP	Mitochondrial permeability transition pore
PCA	Principal component analysis
PCR	Polymerase chain reactions
RyR2	Ryanodine receptor 2
SCWs	Spontaneous Ca waves
SEM	Standard error of the mean
SR	Sarcoplasmic reticulum
SRA	Sequence Read Archive
TLR	Toll-like receptor
WGA	Wheat germ agglutinin
WT	Wild type
