# Supplementary material for: Genetic Inhibition of Mitochondrial Permeability Transition Pore Exacerbates Ryanodine Receptor 2 Dysfunction in Arrhythmic Disease
Source: Cells. 2023 Jan 4;12(2):204. doi: 10.3390/cells12020204 (PMC9856515; doi:10.3390/cells12020204)
Supplement: Supplementary file 1 [file cells-12-00204-s001.zip › Supplementary Figures S1-3.pdf]

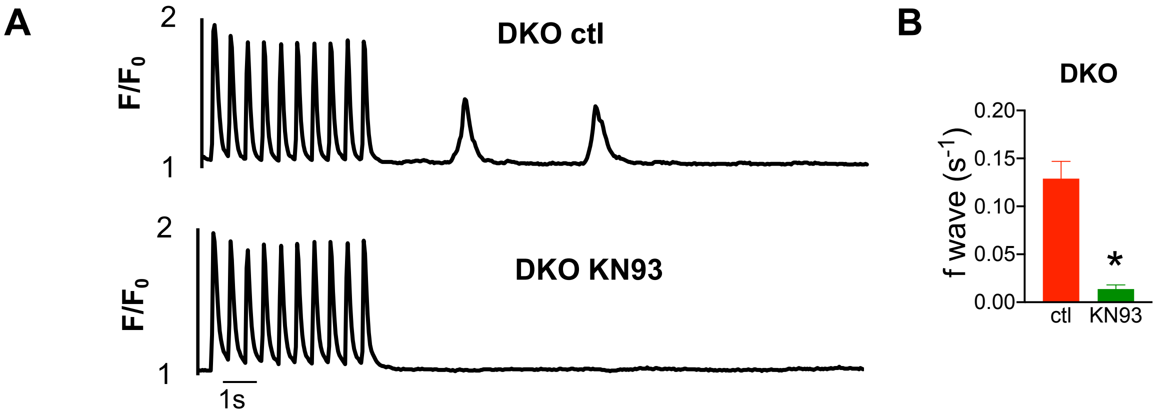

Supplemental Figure S1: The CaMKII inhibitor KN93 reduced the frequency of Ca^{2+} waves in the DKO cells. A, Representative traces of time-dependent fluorescence profiles of Ca^{2+} transient and SCWs in the presence of 100 nM ISO, with or without the CaMKII inhibitor KN93. B, Mean ± SEM of average of frequency of Ca^{2+} waves after pacing at 2 Hz for 5 s, with or without the CaMKII inhibitor KN93 (1μM), (n=30-68 cells from N=3 mice), * $p<0.05$, unpaired Student's t-test between the different groups of cells.

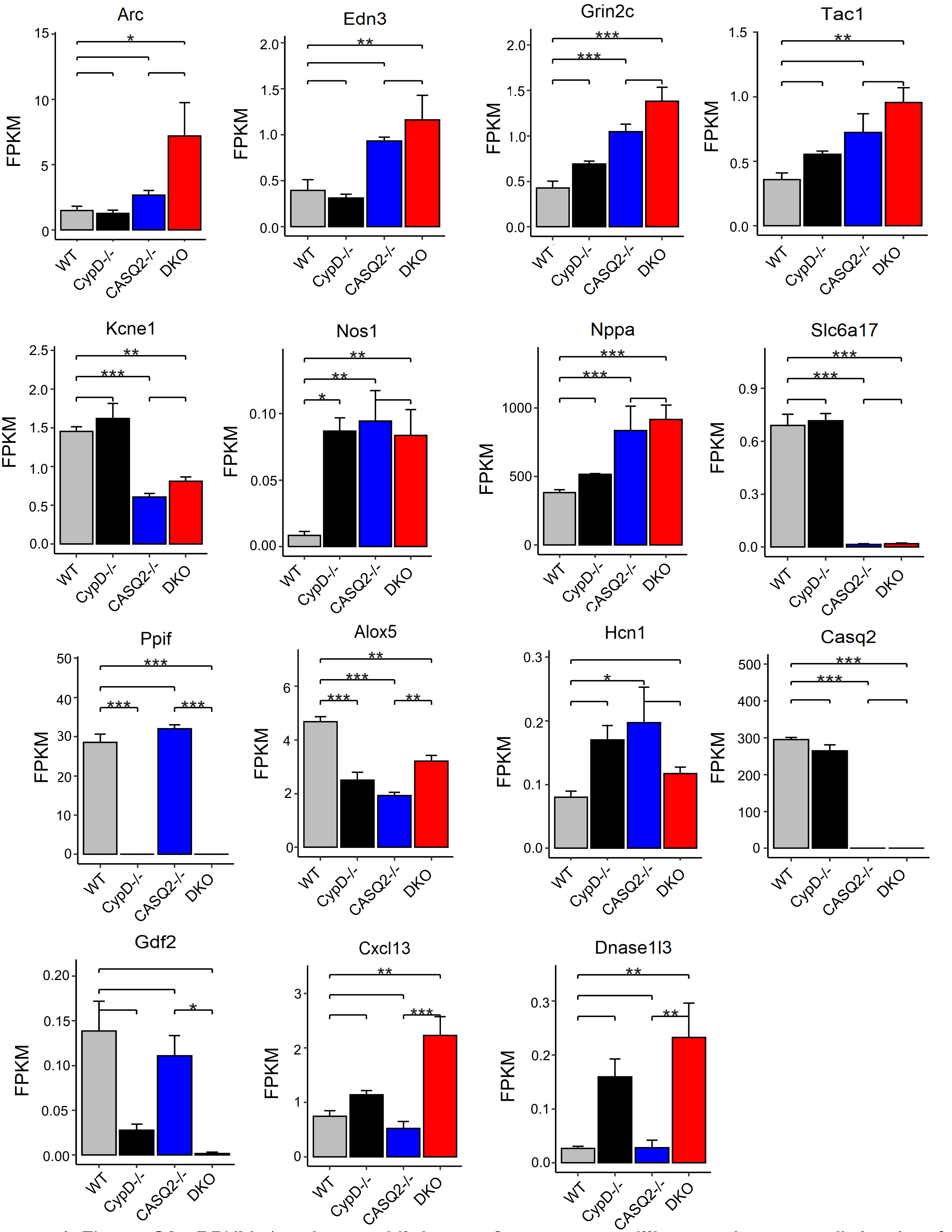

Supplemental Figure S2: RPKM (**reads per kilobase of exon per million reads mapped**) levels of genes involved in pathways regulating ion transport and thus impacting heart rhythm. Statistical analysis was conducted between WT and each of the three mutant groups, as well as between CASQ2-/- and DKO. *, significant difference level at $p_{adj}<0.05$; **, significant difference level at $p_{adj}<0.01$; ***, significant difference level at $p_{adj}<0.001$.

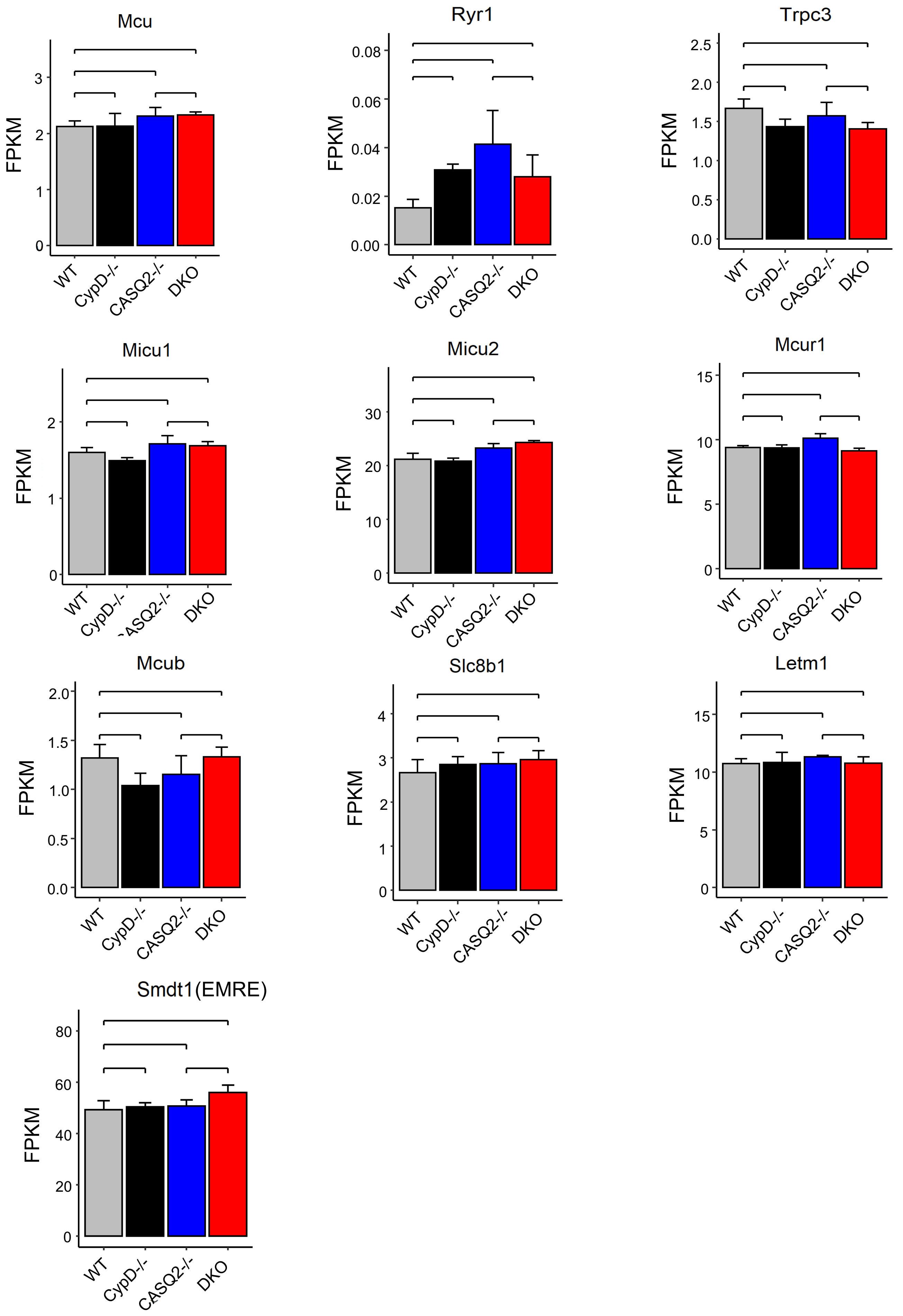

Supplemental Figure S3: RPKM levels of genes involved in mCa^{2+} handling. Statistical analysis was conducted between WT and each of the three mutant groups, as well as between CASQ2-/- and DKO. *, significant difference level at $p_{adj}<0.05$. No significant differences were detected in the comparisons.

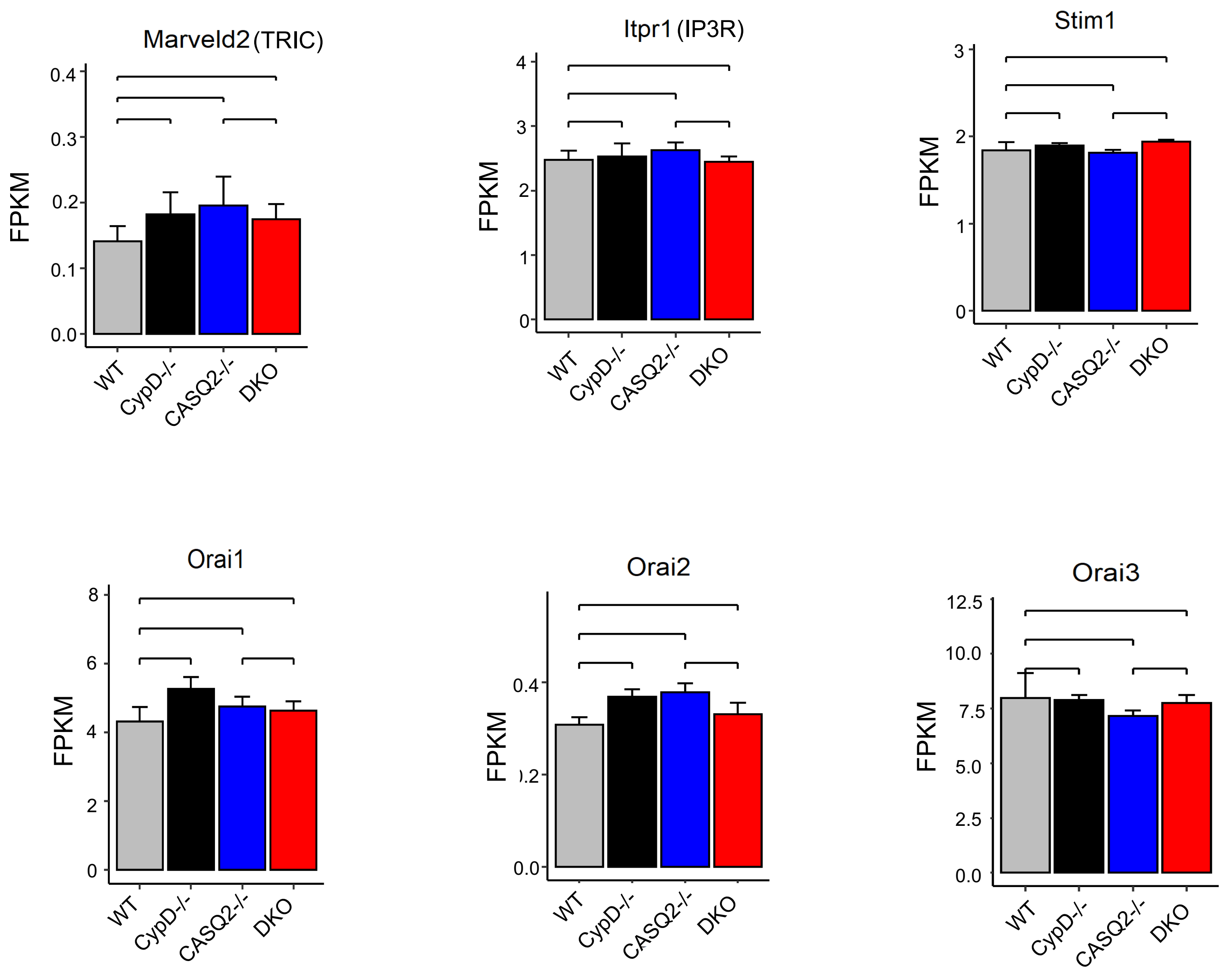

Supplemental Figure S4: RPKM levels of IP3R, TRIC channels and proteins in the SOCE pathway. Statistical analysis were conducted between WT and each of the three mutant groups, as well as between CASQ2-/-and DKO. *, significant difference level at $p_{adj}<0.05$. No significant differences were detected in the comparisons.

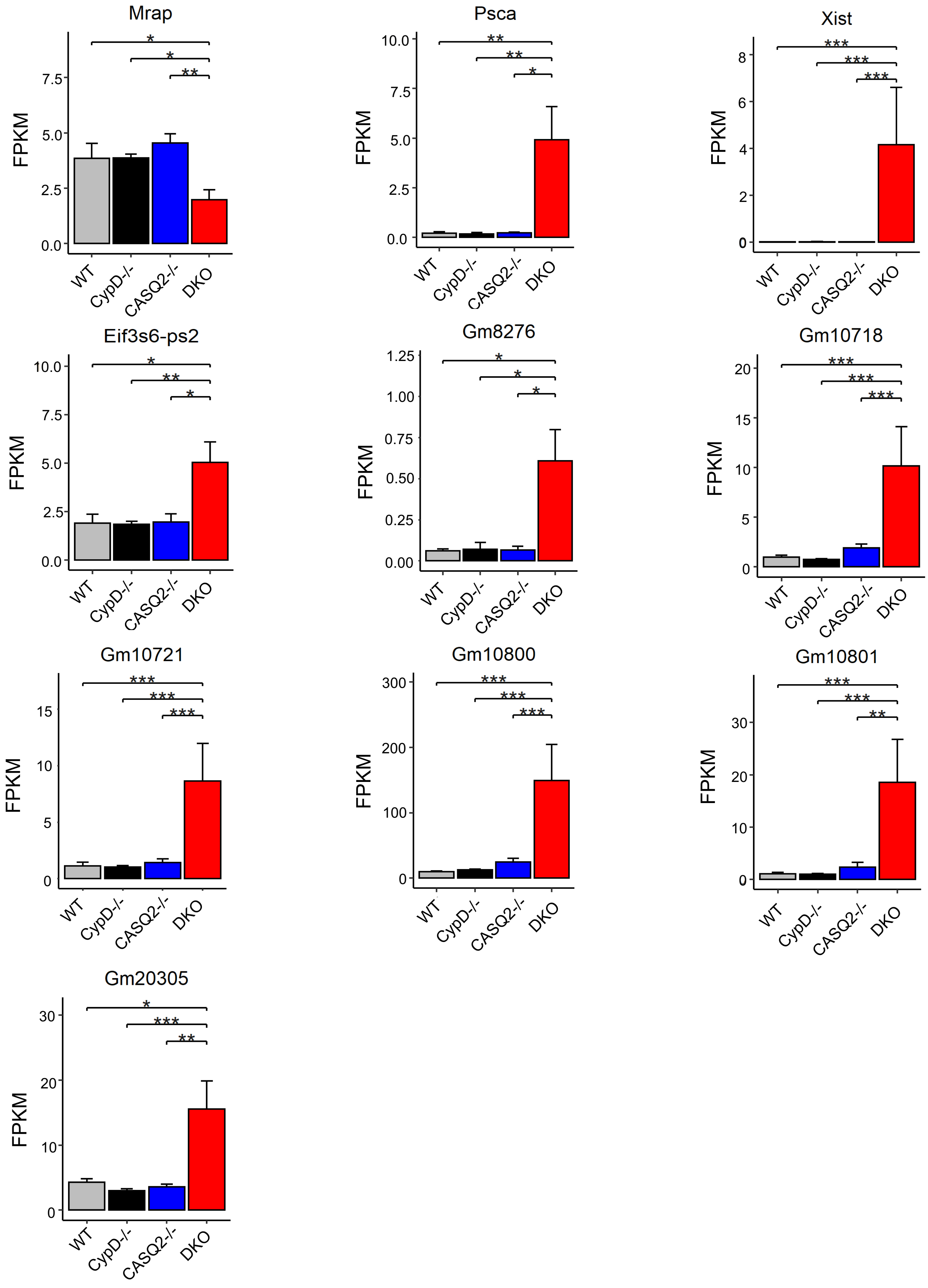

Supplemental Figure S5: RPKM levels of transcripts that were expressed differentially in the DKO group. Statistical analysis were conducted between DKO and three of the control groups. *, significant difference level at $p_{adj}<0.05$; **, significant difference level at $p_{adj}<0.01$; ***, significant difference level at $p_{adj}<0.001$.

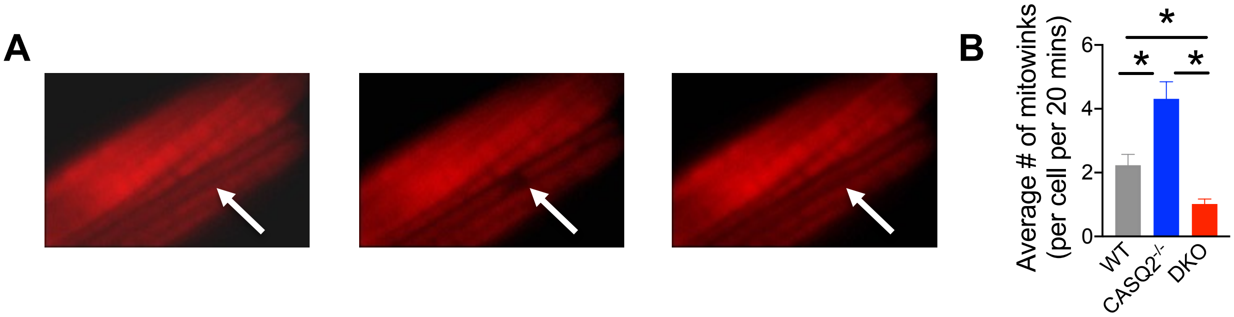

Supplemental Figure S6: the transient opening mode of mPTP was inhibited in the DKO cells. A, Representative image of a MitoWink, whereby a mitochondrion undergoes transient depolarization due to the brief opening of mPTP. B, Mean ± SEM of average number of MitoWinks per cell per 20 min (n=54-64 cells from N=3 mice). * $p<0.05$ one-way ANOVA between groups.

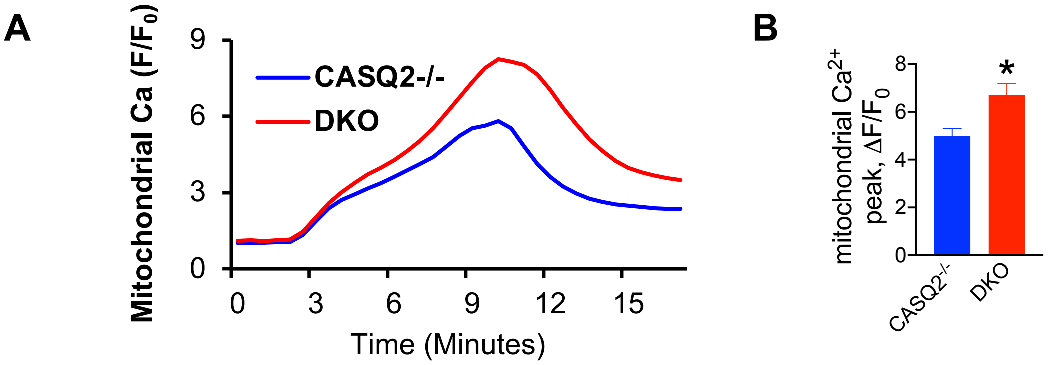

Supplemental Figure S7: Increased mitochondrial Ca^{2+} in DKO. A, Representative mitochondrial Ca^{2+} dynamics in response to 1 Hz pacing and ISO perfusion. B, Mean ± SEM of peak mitochondrial Ca^{2+} signal in response to pacing and ISO perfusion (n=24-38 cells from N=3 mice). * $p<0.05$ unpaired Student's t-test.
